# Channel estimation for reconfigurable intelligent surface-assisted mmWave based on Re‘nyi entropy function

**DOI:** 10.1038/s41598-022-26672-3

**Published:** 2022-12-24

**Authors:** Zaid Albataineh, Khaled F. Hayajneh, Hazim Shakhatreh, Raed Al Athamneh, Muhammad Anan

**Affiliations:** 1 Department of Electronics Engineering, Yarmouk University, Irbid, 21163 Jordan; 2 Telecommunications Engineering Department, Yarmouk University, Irbid, 21163 Jordan; 3 Department of Industrial Engineering, Faculty of Engineering, The Hashemite University, Zarqa, 13133 Jordan; 4 Software Engineering Department, Alfaisal University, 11533 Riyadh, Saudi Arabia

**Keywords:** Engineering, Electrical and electronic engineering

## Abstract

This study focuses on channel estimation for reconfigurable intelligent surface (RIS)-assisted mmWave systems, in which the RIS is used to facilitate base-to-user data transfer. For beamforming to work with active and passive elements, a large-size cascade channel matrix should always be known. Low training costs are achieved by using the mmWave channels’ inherent sparsity. The research provides a unique compressive sensing-based channel estimation approach for reducing pilot overhead issues to a minimum. The proposed technique estimates channel data signals in a downlink for RIS-assisted mmWave systems. The mmWave systems often have a sparse distribution of signal sources due to the spatial correlations of the domains. This distribution pattern makes it possible to use compressive sensing methods to resolve the channel estimation issue. In order to decrease the pilot overhead, which is necessary to predict the channel, the proposed method extends the Re‘nyi entropy function as the sparsity-promoting regularizer. In contrast to conventional compressive sensing techniques, which necessitate an initial knowledge of the signal’s sparsity level, the presented method employs sparsity adaptive matching pursuit (SAMP) techniques to gradually determine the signal’s sparsity level. Furthermore, it introduces a threshold parameter based on the signal’s energy level to eliminate the sparsity level requirement. Extensive simulations show that the presented channel estimation approach surpasses the traditional OMP-based channel estimation methods in terms of normalized mean square error performance. In addition, the computational cost of channel estimation is lowered. Based on the simulations, our approach can estimate the channel well while reducing training overhead by a large amount.

## Introduction

In the era of 5G/6G and the Internet of Things, the network capacity is expected to increase by a factor of 1000 by 2022, allowing for the support of at least 50 billion devices through wireless communications while simultaneously reducing energy consumption^1^. Recently, a possible new technology called a Reconfigurable intelligent Surface (RIS) has been suggested to meet these Quality-of-Service (QoS) requirements. To boost signal strength^2–4^ or to reduce interference and improve security/privacy^5,6^, it is feasible to modify the reflected signal into particular receivers by adjusting the phase of each passive element on the surface in real time with little power consumption.

The RIS is a promising new method for boosting the efficiency of wireless networks^7,8^. The RIS has excellent potential for increasing spectrum efficiency and energy efficiency, especially for millimeter-wave (mmWave) communication systems, which are susceptible to high path loss and link blockage^1,2^. The RIS can “program” the wireless propagation channel by modifying the dissipation and/or phase angle of significant passive reflecting elements. Some of the many areas of ongoing RIS studies include channel modeling^1,4^, beamforming design^5,6^, and hardware testing^9,10^. Intelligent and customizable wireless propagation environments may be realized using many passive reflective devices in the RIS^1–4^. Using a smart controller, each element’s incident signal may be separately reflected with a re-configurable amplitude and phase shift. The reflected signals can be added coherently to the selected receiver by changing the phase shifts of the passive parts. To overcome obstacles that prevent a direct line-of-sight (LOS) connection, the RIS protocol was recently established^5,6^.

There are several advantages to massive MIMO systems, such as focusing a significant amount of energy in three-dimensional space that paves the way for wireless charging and distant sensing and data transfers, which can be achieved using RIS. There are, of course, essential distinctions between massive MIMO and the aforementioned RIS. In order to deliver high data rates for indoor devices through near-field communications, the RIS may be intensively installed in interior locations^11^. It is also cost-effective and energy efficient compared to typical active antenna arrays, which use radio frequency chains and power amplifiers. This makes RIS a potential energy-efficient solution in green communications. Third, unlike typical full-duplex relays, the RIS simply reflects the signal back to the sender. This eliminates thermal noise and self-interference from the received signal. RIS has been extensively studied in a variety of wireless communication systems because of its significant benefits. RIS active and passive beamforming requires precise channel state information (CSI) in order to maximize their potential. Channel estimate for RIS -aided wireless systems has already been done, for example,^9–13^. In^9^, active elements were utilized at the RIS in order to ease channel estimation. These active components can be used in a receive mode to estimate the BS- RIS channel and the RIS-user channel. The wiring or battery power required by RISs with active components makes them unsuitable for many applications. The least squares (LS) estimation approach was presented for RISs with all passive parts in order to estimate up-link cascade channels. There is a problem with the size of the cascade channel. These approaches may need significant training overhead since they do not take advantage of the sparse structure inherent in wireless channels. The low-rank nature of the BS- RIS and RIS -user channels are used to construct a sparse matrix factorization-based channel estimation approach in^12^. The suggested solution necessitates the periodic deactivation of specific passive components. However, implementing the ON/OFF switching is expensive since each RIS element must be controlled separately in terms of amplitude^13^. Academic and industrial research has focused on RISs because of their enticing advantages in offering efficient communications in terms of both energy and spectrum efficiency^11–15^. In order to reflect radio waves without the need for RF chains, a RIS is a thin surface made up of virtually passive and reconfigurable reflecting parts. It is possible to modify the electromagnetic response of the passive elements on the RIS to boost or degrade the signals reflected from a RIS, enhancing or degrading their effectiveness at the target receiver. As can be seen in^16^, these features make it an attractive candidate for inclusion in a wide range of communication systems. Single-cell multiple-output (MIMO) systems^17–21^ and multicell MIMO communications^7^ can all benefit from it, as can SWIPT systems^8,17^, secure communications^22^, mmWave systems^23–25^, and THz systems^22,26^. It can also be used in single-cell MIMO systems.

In the research literature, RIS is being studied for a number of purposes, such as improving physical layer security^22^, extending coverage to users in dead zones^23^, and mitigating co-channel interference when users are close to the cell’s perimeter. Furthermore, the RIS might be employed for simultaneous wireless data and power transfer in an IoT network^24,27^. In the realm of wireless communication networks, the relationship between RIS technology and mmWave hybrid MIMO systems has recently been established^25^. In this work, the authors consider a RIS-enabled hybrid MIMO-OFDM operating in the mmWave spectrum. The advantages and drawbacks of RIS-assisted wireless communications have been explored in recent literature^22,26,28–30^. We emphasize gathering channel status information as one of the many outstanding difficulties. The assumption that the RIS is often made up of passive parts presents a problem since the receiver needs to estimate the cascaded channel using pilots provided by the transmitter through the RIS. The RIS’s phase shift pattern during training is now crucial. Furthermore, the channel estimation issue is complicated by the high number of RIS pieces. In the literature, two distinct methods have been offered. In the first scenario, the RIS is only partially active, consisting of a few active components linked to RF chains for the reception. With some degree of baseband processing already present at the RIS, CSI acquisition is made more accessible. In^31,32^, we have an example of this strategy where compressive sensing is used to estimate the relevant channels. In contrast, the second method, which is the one used in this article, assumes a passive structure in which the RIS accomplishes its goals by returning the impeding waves in accordance with a phase-shift rhythm. For mmWave communications aided by a reconfigurable intelligence surface (RIS), estimating the channel is a significant challenge. Since the pilot overhead in such a system is proportional to the product of the number of base station antennas and the number of RIS components, the number of coefficients of the cascaded channels is unacceptably large. To that end, a handful of studies have discussed the issue of channel estimation and provided varying solutions for the passive RIS scenario. In^33,34^, an optimum RIS phase shift matrix design is discovered, and a minimal variance unbiased estimator is presented. The authors of^35,36^ use sparse representations to suggest a two-stage approach. Authors in^27,34^ describe novel channel estimation based on beam training and ties between massive MIMO and RIS. Using a channel estimate strategy,^36,37^ suggests RIS lessens the blockage issue’s impact on mmWave communications. Working off of compressive sensing (CS) techniques, the authors of^38^ offer a methodology for uplink channel estimation in an RIS-assisted multi-user MIMO system. In^39^, a MIMO system with an RIS is explored; channel estimation is performed using two stages based on the approximate message-passing technique. For a situation where an RIS is helping to connect devices over the internet,^40^ presents a combined channel estimation and active detection using matrix completion and sparse matrix factorization. While^41^ proposes a feasible transmission protocol to achieve channel estimation and passive beamforming,^34,42^ proposes a channel estimation method in which the RIS-UT, BS-UT, and BS-RIS channel models are identified in a two-timescale manner. In^43^, the sequential activation of the RIS components is used to do channel estimation using an on-off method. In order to address the channel estimation issue in a MISO environment with many users, the authors of^44^ suggest a parallel factor model. The vast majority of published RIS-assisted communications studies focus on the MISO scenario when a receiver station has more than one antenna. The last 10 years have seen the successful application of tensor modeling to a wide range of signal processing problems^45–47^, especially in the realm of wireless communications, including the semi-blind channel estimation methods for massive MIMO scenarios^48,49^, channel estimation for cooperative communication systems^27,50,51^, the direction of arrival estimation^52–55^, and, channel estimation based on CS in MIMO communication systems^56^. Most of these publications agree that tensor-based signal processing is superior because it takes full use of the multi-dimensionality of both the broadcast and received signals and the communication channels while also benefiting from the strong uniqueness qualities of tensor decompositions. This paper shows that RIS-assisted MIMO communications may be linked to tensor modeling. In^57^, the authors demonstrate that the received signal is consistent with a parallel factor (PARAFAC) tensor model by assuming a structured time-domain pattern of pilots and RIS phase changes. They present two easy-to-implement techniques for estimating the cascaded MIMO channel by decoupling the transmitter-RIS and RIS-receiver MIMO channels, respectively, using the PARAFAC signal structure in two distinct ways. The first method is a recursive application of the bilinear alternating least squares (BALS) formula, which is derived from the Khatri-Rao factorization (KRF) of the combined BS-RIS and RIS-UT channels. The first method yields a closed-form algebraic solution, whereas the second has looser constraints on the system’s parameters. Both methods improve performance over the direct estimation of the cascaded channel using classical least squares by decoupling the estimation of the two relevant channel matrices. However, this work’s contributions are summed up as follows:

- The goal of this study is to provide a channel estimate for mmWave systems that make use of RIS.
- For the cascaded BS-RIS-user channel, we adopt a sparse reconstruction based on attributes from the Khatri-Rao and Kronecker products. This results in the channel estimation challenge being re-framed as one of recovering sparse signals^5^.
- The proposed technique utilizes an extended version of the Re‘nyi entropy function as the sparsity promoting regularizer in mmWave downlink MIMO systems to cut down on the amount of pilot overhead needed to predict the channel^58^. The proposed technique uses the SAMP methodology^59^ to incrementally calculate the sparsity level and adds the sparsity threshold energy level to do away with the need for the sparsity level, which is a prerequisite for the traditional compressive sensing algorithm.
- The proposed approach is an improvement over earlier ones since it estimates the channel without needing any knowledge of its sparsity in advance.

Many simulations were run to test the efficacy of the proposed approach to channel prediction in RIS-aided mmWave systems. Our simulation results show similar performance for channel estimation and faster convergence times. We present metric comparisons based on the normalized mean square error (NMSE) as a function of signal-to-noise ratio (SNR) and pilot sequence number. Our method significantly reduces training time and effort while increasing confidence in the estimated channel. Throughout this work, we employ the following notation: scalars are represented by lowercase, vectors by italicized lowercase, and matrices by italicized uppercase. $diag(\nu )$ stands for the diagonal matrix associated with the vector $\nu$, whereas $(.)^T$ represents the transposition operator, $(.)^H$ is the Hermitian transpose operator, and $\Vert . \Vert _p$ signifies the $l_p$-norm.

The rest of the paper is organized as the followings: “System model and problem formulation” section shows a short discussion and derivation of the RIS-assisted mmWave systems. “Proposed method” section describes the traditional CS model. “Results” section proposes a novel channel estimate approach based on the Re‘nyi entropy function. “Discussion” and “Conclusions” sections provide the simulation findings and conclusions, respectively.

## System model and problem formulation

We study an RIS-assisted mmWave downlink system in which a RIS is used to enhance data transmission from the BS to a single-antenna user. Assume the RIS is a planar array comprised of *M* reflecting components. *N* antennas are installed on the BS. $G \in {\mathbb {C}}^{(M\times N)}$ represents the channel matrix from the BS to the RIS, and $h_r \in {\mathbb {C}}^{(M\times 1)}$ represents the channel vector the RIS to the user. Without loss of generality, we omit the direct link between the BS and the user. Nonetheless, adding a direct link from the BS to the user to the scenario is simple. A sophisticated controller allows each RIS reflecting device to reflect the incident signal with a reconfigurable phase shift and amplitude^3^.

Also, let us denote that the phase matrix of the RIS as $\phi \equiv diag(\beta _1 e^{j\theta _1},\ldots , \beta _M e^{j\theta _M} )$, where $\beta _m \in [0,1]$ and $\theta _m \in [0,2\pi ]$, $m\in [1,2,\ldots , M]$, represent the amplitude reflection coefficient and phase shift associated with the *m*-th passive element of the RIS, respectively.

The received signal of the user at the *l*-th time instant can be expressed as

1 $$\begin{aligned} y_l=h_r^T \phi _l Gb_l s_l+n_l, \end{aligned}$$

where $s_l$ represents the transmitted symbol at the lth time instant, $b_l$ is the beamforming vector at the *l*-th time instant, and $n_l$ represents the additive white Gaussian noise with zero mean and variance $\sigma ^2$. Now, let us assume that the cascaded channel is denoted by $H \cong diag(h_r^T)G \in {\mathbb {C}}^{M\times N}$. Then the received signal of the user in Eq. (1) can be rewritten as

2 $$\begin{aligned} y_l=v_l^T Hb_l s_l+n_l, \end{aligned}$$

where $v_l^T \cong [e^{j\theta _1},\ldots ,e^{j\theta _M } ]^T\in {\mathbb {C}}^M$, denotes the phase shift vector at the lth time instant.

To that end, mmWave systems may include a large number of reflecting components (*M*) and antennas (*N*). According to studies of mmWave channels in real-world environments^5,14,15,27,32,34,36^, it is possible to significantly minimize training overhead by exploiting their sparse scattering features.

To that end, The BS will have N antennas, while the RIS will have M, and both will be using the uniform linear array (ULA) and uniform planer array (UPA), respectively. Let $G\in {\mathbb {C}}^{M\times N}$ represent the RIS-to-BS channel and let $h \in {\mathbb {C}}^{M\times 1}$ represent the kth-user-to-RIS channel ($k=1,2,\ldots ,K$). *G* is often represented as in^5,60^ using the Saleh-Valenzuela channel model.

3 $$\begin{aligned} G= \sqrt{ \frac{MN}{L_G} } \sum _{l_1}^{l_G} \alpha _{l_1}^G b(\theta _{l_1}^{G_r}, \phi _{l_1}^{G_r}) a(\theta _{l_1}^{G_t}, \phi _{l_1}^{G_t})^T, \end{aligned}$$

where $L_G$ represents the number of paths between the RIS and the BS, $\alpha _{l_1}^G$ denotes the complex gain consisting of path loss for the $l_1$-th path, $\theta _{l_1}^{G_r}$ and $\phi _{l_1}^{G_r}$ represent the azimuth and elevation angle at the BS for the $l_1$-th path, respectively. $\theta _{l_1}^{G_t}$ and $\phi _{l_1}^{G_t}$ denote the azimuth and elevation angle at the RIS for the $l_1$-th path, respectively. The normalized array steering vectors for the BS and RIS are denoted by $b(\theta ,\phi ) \in {\mathbb {C}}^{N\times 1}$ and $a(\theta ,\phi )\in {\mathbb {C}}^{M\times 1}$, respectively.

Nevertheless, let $h_{r,k}\in {\mathbb {C}}^{N\times 1}$ represent the channel among *k*-th user and the RIS, and it is expressed as

4 $$\begin{aligned} {h_{r,k}} = \sqrt{\frac{M}{{{L_{r,k}}}}} \mathop \sum \limits _{{l_2} = 1}^{{L_{r,k}}} \alpha _{{l_2}}^{r,k}a\left( {\vartheta _{{l_2}}^{r,k},\varphi _{{l_2}}^{r,k}\;} \right) , \end{aligned}$$

where ${L_{r,k}}$ represents the number of paths between the *k*-th user and the RIS, $\alpha _{{l_2}}^{r,k}$ represents the complex gain consisting of path loss for the ${l_2}$-th path, $\vartheta _{{l_2}}^{r,k}$ and $\varphi _{{l_2}}^{r,k}$ represent the azimuth and elevation angles of the ${l_2}$th path, respectively.

For a typical $M = {M_1} \times {M_2}$ planar antenna, $a\left( {\vartheta ,\varphi } \right)$ can be expressed as

5 $$\begin{aligned} a\left( {\vartheta ,\varphi } \right) = \frac{1}{{\sqrt{M} }}\left[ {{e^{{{{ - j2\pi dsin\left( \vartheta \right) {\textrm{cos}}\left( \varphi \right) {n_1}}}/ {\lambda }}}}} \right] \otimes \left[ {{e^{{{{ - j2\pi dsin\left( \varphi \right) {n_2}}} / {\lambda }}}}} \right] \end{aligned}$$

where ${n_1} = \left[ {0,\; \ldots ,\;{M_1} - 1} \right]$ and ${n_2} = \left[ {0,\; \ldots ,\;{M_2} - 1} \right]$, $\lambda$ denotes the wavelength of the carrier signal, and $d$ stands for the typical antenna spacing that satisfies $d = {\lambda } /{2}$. Let ${H_k} = Gdiag\left( {{h_{r,k}}} \right) \in {{\mathbb {C}}^{M \times N}}$ represents the cascaded channel for the kth user. Then, ${H_k} \in {{\mathbb {C}}^{M \times N}}$ can be decomposed as

6 $$\begin{aligned} H_k=U_M \check{H}_k U_N^T, \end{aligned}$$

where $\check{H}_k$ denotes the $M \times N$ angular cascaded channel, ${U_M}$ and ${U_N}$ denote the $M \times M$ and $N \times N$ unitary matrices^9^. The sparsity is shown by the fact that the angular cascaded channel $\check{H}_k$ contains only a small number of non-zero elements due to the low scattering at the BS and RIS.

The cascaded channel estimation problem is the core of this paper. In order to apply the standard orthogonal pilot transmission method for uplink channel estimation, all users must send the known pilot symbols to the BS through the RIS at different$\;Q$ time frames. In particular, after eliminating the effects of the direct channel, the effective received signal ${y_{k,q}} \in {{\mathbb {C}}^{N \times 1}}$ of the $k$-th user in the $q$-th ($q = 1,\;2,\; \ldots ,\;Q$) timeslot may be expressed as

7 $$\begin{aligned} {y_{k,q}} = Gdiag\left( {{h_{r,q}}} \right) {\theta _q}{s_{k,q}} + {w_{k,q}}, \end{aligned}$$

where ${s_{k,q}}$ denotes the pilot symbol consigned by the $k$-th user, ${\theta _q} = {\left[ {{\theta _{q,1}},\;{\theta _{q,2}},\; \ldots ,\;{\theta _{q,M}}\;} \right] ^T}$ represents the $M \times 1$ reflecting vector for the RIS, ${\theta _{q,n}}$ denotes the reflected RIS coefficient of the $n$-th component. Furthermore, the ${w_{k,q}} \in {{\mathbb {C}}^{N \times 1}}$ represents the noise with ${\sigma ^2}$ representing noise power, i.e., ${w_{k,q}} \sim {\mathscr {N}}(0,\,\sigma ^{2} I_N)$ .

According to the cascaded channel ${H_k} = Gdiag\left( {{h_{r,k}}} \right)$, we can rewrite Eq. (7) as

8 $$\begin{aligned} {y_{k,q}} = {H_k}{\theta _q}{s_{k,q}} + {w_{k,q}}. \end{aligned}$$

After $Q$ time slots of pilot transmission, we can obtain the $N \times Q$ overall measurement matrix ${Y_k} = \left[ {{y_{k,1}},{y_{k,2}}, \ldots ,\;{y_{k,Q}}\;} \right]$ by assuming ${s_{k,q}} = 1$.

The $N \times Q$ global measurement matrix ${Y_k} = \left[ {{y_{k,1}},{y_{k,2}}, \ldots ,\;{y_{k,Q}}\;} \right]$ may be derived from the data collected during the $Q$ time slots of the pilot broadcast. where we assume that the ${s_{k,q}} = 1$ as follows:

9 $$\begin{aligned} {Y_k} = {H_k}{\varvec{\phi }} + {W_k}, \end{aligned}$$

where $${\varvec{\phi }} = \left[ {{\theta _1},{\theta _2}, \ldots ,\;{\theta _Q}\;} \right] $$ and $${W_k} = \left[ {{w_{k,1}},{w_{k,2}}, \ldots ,\;{w_{k,Q}}\;} \right] $$.

By substituting, one can rewrite equation Eq. (9) as

10 $$\begin{aligned} {Y_k} = U_M \check{H}_k U_N^T {\varvec{\phi }} + {W_k}. \end{aligned}$$

where $U_M$ and $U_N$ are the unitary matrices as in (6).

Let us assume that the ${{\tilde{Y}}_k} = {\left( {U_M^H{Y_k}} \right) ^H}$ as the $Q \times N$ measurement matrix, and ${{\tilde{W}}_k} = {\left( {U_M^H{W_k}} \right) ^H}$ as the $Q \times N$ noise matrix. One can rewrite Eq. (10) as a compressed sensing model:

11 $$\begin{aligned} {{\tilde{Y}}_k} = {\varvec{\tilde{\phi }}}{\tilde{H}}_k^H + {{\tilde{W}}_k}, \end{aligned}$$

where ${\varvec{\tilde{\phi }}} = {\left( {U_N^H{\varvec{\phi }}} \right) ^H}$ denotes the $Q \times N$ mixing matrix. Based on $x$, we may utilize traditional CS techniques like the OMP algorithm to predict the angular cascaded channel for each user $k$.

To that end, we have investigated sparse recovery techniques. In order to handle the underdetermined issue, sparse recovery algorithms are divided into two types based on the CS theory. First, convex optimization methods-based l 1-norms approaches. Others are based on greedy algorithms. This study uses the recently developed greedy method, which offers low computational cost and the same guarantees as the best optimization-based algorithms^17^.

## Proposed method

In this part, we propose a novel adaptive channel estimation technique using the Re‘nyi entropy function and SAMP algorithms^58^. One can reconstruct the unknown *K*-Sparse vector ${\tilde{h}}_k$ in Eq. (11) by bargaining the sparsest estimate of $h_k$ as follows:

12 $$\begin{aligned} \text {min}_{\tilde{h_k}} \parallel \tilde{h_k} \parallel _{l_0}~~ \text {s. t.} \parallel {\tilde{ {y}_k}}- {\varvec{{\tilde{\phi }}}} {\tilde{h_k}} \parallel _{l_2} \le \varepsilon . \end{aligned}$$

where $\varepsilon$ is the $l_2$ norm error tolerance.

In the information theory^22,58^, entropy quantifies a system’s diversity, uncertainty, or randomness. Re‘nyi entropy^26,58^ is defined with respect to the probability distribution ${\mathscr {P}}({\mathscr {H}})=\{{\mathscr {P}}(h_1), {\mathscr {P}}(h_2), \ldots , {\mathscr {P}}(h_K) \}$ of some random variable ${h}_{{k}}$ as follows:

13 $$\begin{aligned} H_\alpha ({\mathscr {H}})={{ \;}}\frac{1}{{1 - \alpha }}{{ \;}}\log \left( {{{ \;\;}}\mathop {\sum }\nolimits _{i = 1}^N {{\left( {{{ \;}}{\mathscr {P}}(h_i){{ \;}}} \right) }^\alpha }{{ \;\;}}} \right) {{ \;}} {{ \;}} \end{aligned}$$

where $\alpha \ge 0$ and $\alpha \ne 1$. ${H_\alpha }\left( {\mathscr {H}} \right)$ represents the uncertainty of the random variable ${\mathscr {H}}$. The degree to which the distribution ${\mathscr {P}}$ is sparse may be quantified using the concept of entropy. On the other hand, one can express the discrete probability distribution ${\mathscr {P}}$ as:

14 $$\begin{aligned} h_k \rightarrow {{ \;}}\left[ {\frac{{|{h_1}{|^p}}}{{h_p^p}}{{ \;\;\;}}, \ldots  ,{{ \;}}\frac{{|{h_K}{|^p}}}{{h_p^p}}{{ \;}}} \right] , \end{aligned}$$

where $p > 0$. Then we plug Eq. (14) into (13) to obtain the generalized Re‘nyi entropy function:

15 $$\begin{aligned} {{\textrm{h}}_{{\mathrm{p,\;}}\alpha }}\left( h_k \right) = {{ \;}}\frac{1}{{1 - \alpha }}{{ \;}}\log \left( {{{ \;\;}}\mathop \sum \nolimits _{i = 1}^N {{\left( {{{ \;}}\frac{{{{\left| {h_i} \right| }^p}}}{{\left| {\left| h_k \right| } \right| _p^p}}{{ \;}}} \right) }^\alpha }{{ \;\;}}} \right) {{ \;}} {{ \;}} \end{aligned}$$

which measures the sparsity of ${\tilde{h_k}}$. This motivates us to use the REF as a regularizer in sparse signal recovery problems (12):

16 $$\begin{aligned} \begin{array}{rrclcl} \displaystyle \min _{\tilde{h_k}}&{\left| {\left| {\tilde{ {y}_k}}- {\varvec{{\tilde{\phi }}}}{\tilde{h_k}} \right| } \right| _2^2} + \lambda h_{p, \alpha }({\tilde{h_k}}) \end{array} \end{aligned}$$

Minimizing the optimization problem Eq. (16) promotes sparsity in the recovered solutions. As shown in^28^, minimizing REF $h_{p, \alpha }({\tilde{h_k}})$ in an orthant ${\mathbb {O}}$ of the Euclidean space ${\mathbb {R}}^K$ determines the solution of the boundary of this orthant. The different values of *p* and $\alpha$ affect the performance of sparse signal recovery. Choosing the optimal values of *p* and $\alpha$ to ensure the best performance of the sparse signal recovery task.

The proposed REF $h_{p, \alpha }({\tilde{h_k}})$ is nonconvex and nonsmooth, and as we mentioned before, we can obtain sparse solutions by minimizing $h_{p, \alpha }({\tilde{h_k}})$ as in^5^. To solve the nonconvex optimization problem Eq. (16), we first use the quadratic approximation of the data fidelity term $f({\tilde{h_k}})={\left| {\left| {\tilde{ {y}_k}}- {\varvec{{\tilde{\phi }}}}{\tilde{h_k}} \right| } \right| _2^2}$ in ($t+1$)-th iteration based on ${\tilde{h_k}}^{(t)}$ from the previous *t*-th iteration^22^:

17 $$\begin{aligned} \begin{array}{rrclcl} f({\tilde{h_k}})={\left| {\left| {\tilde{ {y}_k}}- {\varvec{\tilde{\phi }}}{\tilde{h_k}} \right| } \right| }_2^2\\ \le f\left( {{{\tilde{h_k}}^{\left( t \right) }}} \right) + \langle {\tilde{h_k}} - {{\tilde{h_k}}^{\left( t \right) }},\nabla {{ \;}}f\left( {{{\tilde{h_k}}^{\left( t \right) }}} \right) \rangle + \frac{\kappa }{2}\left| \left| {\tilde{h_k}} - {{\tilde{h_k}}^{\left( t \right) }}\right| \right| _2^2\\ = f\left( {{{\tilde{h_k}}^{\left( t \right) }}} \right) - \frac{1}{{2\kappa }} \left| \left| \nabla f({{\tilde{h_k}}^{\left( t \right) }})\right| \right| _2^2 + \frac{\kappa }{2} \left| \left| x - \left( {{{\tilde{h_k}}^{\left( t \right) }} - \frac{1}{\kappa }\nabla f({{\tilde{h_k}}^{\left( t \right) }})} \right) \right| \right| _2^2\\ = {{ \;}}o{{ \;}}{{\tilde{h_k}}^{\left( t \right) }} + {{ \;}}\frac{\kappa }{2} \left| \left| {\tilde{h_k}} - \left( {{{\tilde{h_k}}^{\left( t \right) }} - \frac{1}{\kappa }\nabla f({{\tilde{h_k}}^{\left( t \right) }})} \right) \right| \right| _2^2, \end{array} \end{aligned}$$

where $\nabla f({{\tilde{h_k}}^{\left( t \right) }}) = 2\left( {{{\varvec{\tilde{\phi }}}^T}{\varvec{\tilde{\phi }}}{{\tilde{h_k}}^{\left( t \right) }} - {{\varvec{\tilde{\phi }}}^T}{\tilde{y_k}}} \right) {{ \;}}$is the gradient of the $f({{\tilde{h_k}}^{\left( t \right) }})$, $o{{ \;}}{{\tilde{h_k}}^{\left( t \right) }}$can be ignored since it is a constant depending on${{ \;}}{{\tilde{h_k}}^{\left( t \right) }}$, $\kappa$ is the Lipschitz constant of the gradient $\nabla f({{\tilde{h_k}}^{\left( t \right) }})$, $\kappa = 2\left( {{{\varvec{\tilde{\phi }}}^T}{\varvec{\tilde{\phi }}}} \right)$ is the smallest value of $\kappa$. The backtracking strategy can be used to find $\kappa$ if it is difficult to compute^22^. Using Eq. (17) the optimization problem Eq. (16) becomes:

18 $$\begin{aligned} \begin{array}{rrclcl} \displaystyle \min _{{\tilde{h_k}}}&{{ \;\;}}\frac{\kappa }{2} \left| \left| {\tilde{h_k}} - \left( {{{\tilde{h_k}}^{\left( t \right) }} - \frac{1}{\kappa }\nabla f({{\tilde{h_k}}^{\left( t \right) }})} \right) \right| \right| _2^2 + \lambda {{ \;}}{{\textrm{h}}_{{\mathrm{p,\;}}\alpha }}\left( {\tilde{h_k}} \right) {{ \;\;}} \end{array} \end{aligned}$$

To solve Eq. (18), we need to solve the nonconvex function ${{\textrm{h}}_{{\mathrm{p,\;}}\alpha }}\left( {\tilde{h_k}} \right)$. However, we replace ${{\textrm{h}}_{{\mathrm{p,\;}}\alpha }}\left( {\tilde{h_k}} \right)$ with its first-order approximation with respect to $\left| {{{\tilde{h_k}}^{\left( t \right) }}} \right|$:

19 $$\begin{aligned} {{\textrm{h}}_{{\mathrm{p,\;}}\alpha }}\left( {\tilde{h_k}} \right) \approx {{\textrm{h}}_{{\mathrm{p,\;}}\alpha }}\left( {\left| {{{\tilde{h_k}}^{\left( t \right) }}} \right| } \right) + \left \langle\left| {\tilde{h_k}} \right| - |{{\tilde{h_k}}^{\left( t \right) }}|,{{ \;}}\nabla {{ \;}}{{\textrm{h}}_{{\mathrm{p,\;}}\alpha }}\left( {\left| {{{\tilde{h_k}}^{\left( t \right) }}} \right| } \right) \right\rangle \end{aligned}$$

where $\nabla {{ \;}}{{\textrm{h}}_{{\mathrm{p,\;}}\alpha }}\left( {\left| {{{\tilde{h_k}}^{\left( t \right) }}} \right| } \right)$ is the gradient with respect to $|{{ \;}}{{\tilde{h}}_i}|$. To avoid $\log {{ \;}}0$ when computing $\nabla {{ \;}}{{\textrm{h}}_{{\mathrm{p,\;}}\alpha }}\left( {\left| {{{\tilde{h_k}}^{\left( t \right) }}} \right| } \right)$, we add positive value $\delta = 1{e^{ - 12}}$ to ${{ \;}}|{\tilde{h}}_i^{\left( t \right) }|$. Equation (17), after ignoring the constants, becomes:

20 $$\begin{aligned} \begin{array}{rrclcl} \displaystyle \min _{{\tilde{h_k}}}&{{ \;\;}}\frac{\kappa }{2} \left| \left| {\tilde{h_k}} - \left( {{{\tilde{h_k}}^{\left( t \right) }} - \frac{1}{\kappa }\nabla f({{\tilde{h_k}}^{\left( t \right) }})} \right) \right| \right| _2^2 + \lambda \left\langle {{ \;\;}}\left| {\tilde{h_k}} \right| ,{{ \;}}\nabla {{ \;}}{{\textrm{h}}_{{\mathrm{p,\;}}\alpha }}\left( {\left| {{{\tilde{h_k}}^{\left( t \right) }}} \right| } \right) {{ \;\;\;\;}} \right\rangle \end{array} \end{aligned}$$

The above equation is a reweighted ${l_1}$-norm minimization problem. We can have the solution of Eq. (20) using the soft thresholding function:

21 $$\begin{aligned} {\tilde{h}}_i^{\left( {t + 1} \right) } = {{ \;}}\Gamma {{{ \;}}_{\frac{{\lambda {{ \;}}}}{\kappa }{{ \;}}{{\textrm{h}}_{{\mathrm{p,\;}}\alpha }}\left( {\left| {{{\tilde{h}}_i}^{\left( t \right) }} \right| } \right) }}{{ \;\;}}\left( {{{ \;}}{{\tilde{h}}_i}^{\left( t \right) } - \frac{1}{\kappa }\nabla f({{\tilde{h}}_i}^{\left( t \right) }}) \right) \end{aligned}$$

where the soft shrinkage operator is defined as:

22 $$\begin{aligned} {\Gamma _\eta }\left( {\tilde{h_k}} \right) = {{ \;}}\left\{ {\begin{array}{*{20}{c}}{0\;\;\;\;\;\;\;\;\;\;\;\;\;\;\;\;\;\;\;\;\;\;\;\;\;\;\;\;\;\;\;\;\;\;\;\;\;\;\;if\;\left| {\tilde{h_k}} \right| \le \eta }\\ {\left( {\left| {\tilde{h_k}} \right| - {{ \;}}\eta } \right) \cdot sign\left( x \right) \;\;\;\;\;if\;\left| {\tilde{h_k}} \right| > \eta }\end{array}{{ \;\;\;\;\;\;\;\;\;\;\;\;\;\;\;\;\;\;\;\;\;\;\;}}} \right. \end{aligned}$$

where $\eta \ge 0$ is the threshold value. The ISTA algorithm that solves the convex problem is attractive due to its simplicity, but it has been recognized as a slow method^21^. However, the FISTA algorithm proposed to speed up the convergence of ISTA, it has a convergence rate of${{ \;}}O\left( {\frac{1}{{{K^2*t^2}}}} \right)$^22^, where $t$ denotes the iteration index. Here we adapted the FISTA algorithm as the accelerated proximal gradient method (APG)^29^, which is proposed to solve the nonconvex and nonsmooth problems. To solve Eq. (16) assuming ${F_{p,\alpha }}\left( {\tilde{h_k}} \right) = f\left( {\tilde{h_k}} \right) + \lambda {{ \;}}{{\textrm{h}}_{{\textrm{p,}}\alpha }}\left( {\tilde{h_k}} \right)$, then we have the following steps:

23 $$\begin{aligned} {n^{\left( t \right) }}= & {} {{ \;}}{{\tilde{h_k}}^{\left( t \right) }} + {{ \;}}\frac{{{h^{\left( {t - 1} \right) }}}}{{{h^{\left( t \right) }}}}{{ \;}}\left( {{{ \;}}{m^{\left( t \right) }} - {{\tilde{h_k}}^{\left( t \right) }}{{ \;}}} \right) + {{ \;}}\frac{{{h^{\left( {t - 1} \right) }}{{ \;\;}} - 1}}{{{h^{\left( t \right) }}}}{{ \;\;}}({{ \;}}{{\tilde{h_k}}^{\left( t \right) }} - {{\tilde{h_k}}^{\left( {t - 1} \right) }}) \end{aligned}$$

24 $$\begin{aligned} {m^{\left( {t + 1} \right) }}= & {} {{ \;}}pro{x_{\frac{\lambda }{{k{{ \;}}}}{h_{p,\alpha }}}}{{ \;\;\;\;}}\left( {{ \;\;}}{n^{\left( t \right) }} - {{ \;}}\frac{1}{\kappa }{{ \;\;}}\nabla f{{ \;}}\left( {{n^{\left( t \right) }}} \right) \right) \end{aligned}$$

25 $$\begin{aligned} {l^{\left( {t + 1} \right) }}= & {} {{ \;}}pro{x_{\frac{\lambda }{{k{{ \;}}}}{h_{p,\alpha }}}}{{ \;\;\;\;}}\left( {{ \;\;}}{{\tilde{h_k}}^{\left( t \right) }} - {{ \;}}\frac{1}{\kappa }{{ \;\;}}\nabla f{{ \;}}\left( {{{\tilde{h_k}}^{\left( t \right) }}} \right) \right) \end{aligned}$$

26 $$\begin{aligned} {h^{\left( {t + 1} \right) }}= & {} {{ \;}}\frac{{1 + {{ \;}}\sqrt{4{{ \;}}{{\left( {{{ \;}}{h^{\left( t \right) }}} \right) }^2} + 1{{ \;}}} }}{2} \end{aligned}$$

27 $$\begin{aligned} {{\tilde{h_k}}^{\left( {t + 1} \right) }}= & {} {{ \;}}\left\{ {\begin{array}{*{20}{c}}{{m^{\left( {t + 1} \right) }}\;\;\;\;\;\;\;\;\;\;\;\;\;\;\;\;\;\;\;\;\;\;\;\;\;\;\;\;\;\;if\;{F_{p,\alpha }}\;\left( {{m^{\left( {t + 1} \right) }}} \right) \; \le \;\;{F_{p,\alpha }}\;\left( {{l^{\left( {t + 1} \right) }}} \right) \;\;}\\ {{l^{\left( {t + 1} \right) }}\;\;\;\;\;\;\;\;\;\;\;\;\;\;\;\;\;\;\;\;\;\;\;\;\;\;\;\;\;\;\;\;\;\;\;\;\;otherwise.\;\;\;\;\;\;\;\;\;\;\;\;\;\;\;\;\;\;\;\;\;\;\;\;\;\;\;\;\;}\end{array}{{ \;}}} \right. \end{aligned}$$

when ${\tilde{h_k}}^{(t+1)}$ reaches convergence or after a certain number of iterations have passed, the iterations are stopped. The proposed method may regain its original sparsity level via a multi-stage iterative process, as specified by the SAMP method. Meanwhile, the threshold set-value is included to guard against the sparsity level *K* being incorrectly estimated by the difference in reconstructed signals’ energy levels. Our proposed approach employs a halting criterion previously utilized in^59,61^ to make use of a useful physical aspect of wireless channels. The criterion for selecting a cutoff point is $\rho _c=c\sigma _n^2$, where c is an optimal coefficient (found to be $c = 2$) and $\sigma _n^2$ is the noise variance (obtained on the receiver side), as described in^62^. Algorithm 1 is a representation of the generated pseudocode.
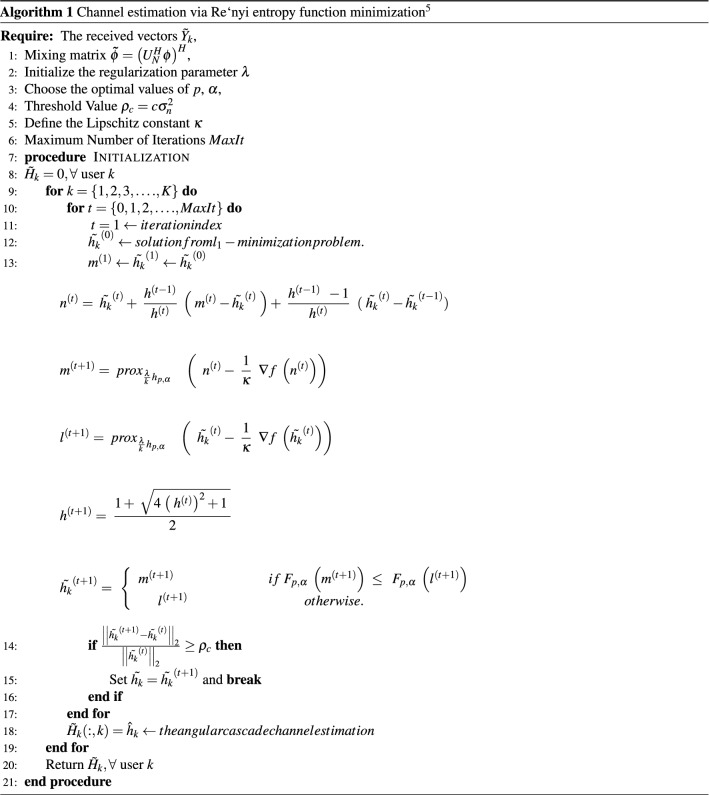


We use the solution from $l_1$-norm minimization as the initializer to ensure the best performance for the proposed algorithm. Additionally, the optimal regularization parameter ($\lambda$) is essential to the success of sparse signal reconstruction. The optimal value of $\lambda$ for noisy recovery depends on the noise level. The proposed Re‘nyi entropy function minimization is in Algorithm 1.

## Results

**Figure 1:**
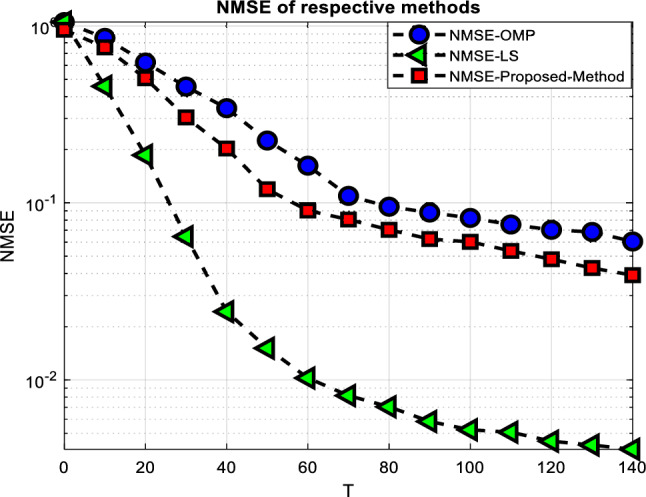
NMSE performance versus the pilot overhead *T*.

Here we show the results of simulations we did to evaluate the performance of our presented channel estimation approach. We evaluate the proposed method in relation to the prevalent CS-based strategy^3^. In the traditional CS-based system, the sparse cascaded channel is estimated using the OMP method. We also employ the Oracle LS scheme as a reference, assuming full knowledge of the supports for all sparse channels. We assume that the BS uses an $N\; = \;16$ antenna ULA and that the RIS employs a $M\; = \;8 \times \;8$ passive reflecting component UPA. Discrete phase shifts of the RIS are taken into account to determine which values between $\frac{{ - 1}}{{\sqrt{M} }}$ and $\frac{{ 1}}{{\sqrt{M} }}$ will make up the RIS reflecting matrix^32^.Based on the recommendations in^23^, we ran our simulations with ${N_G} = \;64$ and ${M_{Gx}} = {M_{Gy}}\; = \;32$. On top of that, we assume a Rician channel with both line-of-sight and non-line-of-sight components^22,24^. A $13.2\;dB$ Rician factor is specified in^24^. For example, if there are three pathways between the RIS and the BS, then there are three paths from the kth user to the RIS, and so on. The AoA and AoD parameters are expected to be evenly generated from $\left[ { - \pi /2,\;\pi \;/2} \right]$, and the spatial angles are considered to be on a quantized grid. NMSE and ARSPR are used as performance metrics. In Fig. 1, we see how the NMSE performs as a function of the pilot overhead, or the total number of time slots T devoted to pilot transmission. Figure 1 shows that compared to the OMP-based technique, the proposed method has less pilot overhead while achieving the same estimate accuracy.

**Figure 2:**
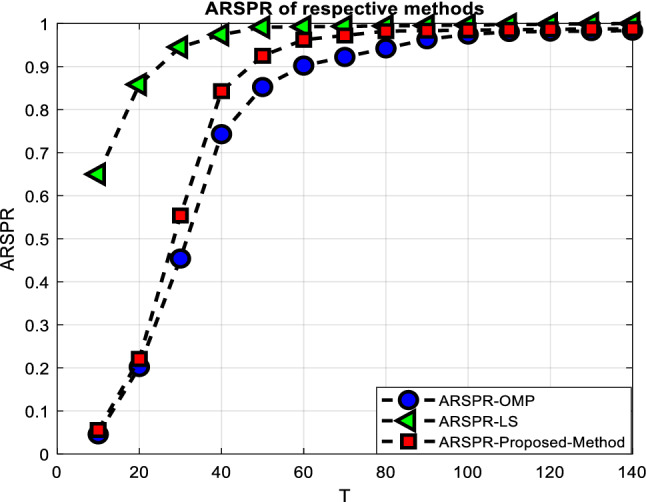
ARSPR performance versus the pilot overhead *T*.

Figure 2 displays the ARSPRs of the relevant algorithms as a function of $T$, with the signal-to-noise ratio (SNR) fixed at $12\;dB$. Figure 2 demonstrates that in realistic scenarios, such as $T\; > \;60$, our presented method may deliver performance (in terms of ARPSR) equivalent to the Oracle LS estimator.

**Figure 3:**
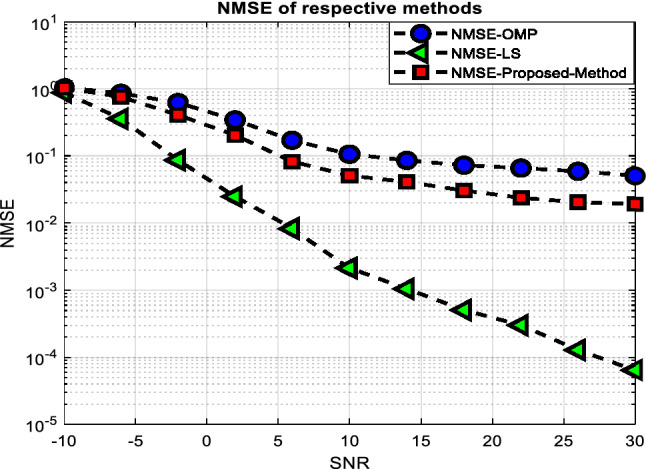
NMSE performance versus the SNR.

**Figure 4:**
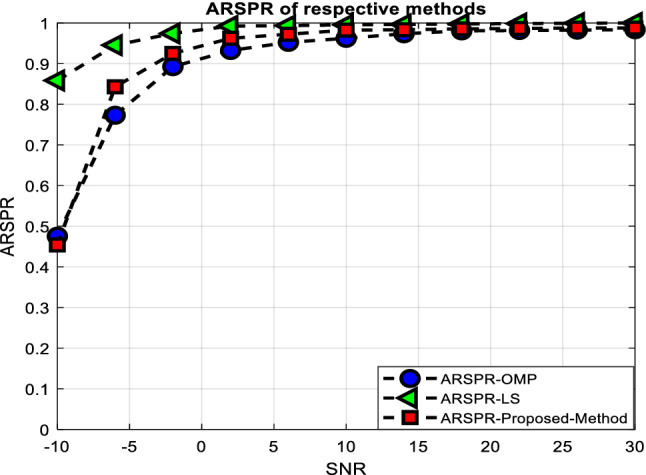
ARSPR performance versus the SNR.

The NMSEs and ARSPRs versus the SNR are shown in Figs. 3 and 4, respectively, with T set to 120. From Fig. 3, one can see that the proposed method improved the system’s performance, given the lowest NMSE concerning OMP based method. Also, Fig. 4 demonstrates that in real-world conditions, such as SNR> 0, our provided method may deliver performance (in terms of ARPSR) equivalent to the Oracle LS estimator.

## Discussion

Within RIS-assisted wireless communication systems, we devised a low-overhead channel estimation technique presented in this article. In particular, the sparsity model of the angular cascaded channels in the mmWave system was the first thing that we investigated and studied. Following the presentation of this sparsity model, we subsequently offered a solution for reducing the pilot overhead that was based on the Re‘nyi entropy function. The simulation results reveal that the suggested approach requires less pilot overhead than the OMP algorithm, which is a significant benefit. In the future, we are going to apply the approach that was provided to the issue of super-resolution channel estimation. We are going to do this by assuming that the channel angles are continuous in actual use.

## Conclusions

We examined the topic of combined beamforming design and channel estimate for RIS-assisted mmWave systems. Utilizing the intrinsic sparse nature of the cascade channel for the RIS-assisted mmWave systems, we developed a compressed sens-ing-based channel estimation approach using the Re‘nyi entropy method. According to simulation findings, our presented scheme can yield a realistic channel estimate and significantly lower training overhead.

## Data Availability

The data used during the current study available from the corresponding author on reasonable request.
